# Radical-Scavenging Activity of Dietary Phytophenols in Combination with co-Antioxidants Using the Induction Period Method

**DOI:** 10.3390/molecules161210457

**Published:** 2011-12-15

**Authors:** Yoshinori Kadoma, Seiichiro Fujisawa

**Affiliations:** 1 Institute of Biomaterials and Bioengineering, Tokyo Medical and Dental University, Kanda-surugadai, Chiyoda-ku, Tokyo 101-0062, Japan; 2 Meikai University School of Dentistry, Sakado, Saitama 350-0283, Japan

**Keywords:** dietary phytophenols, combination, co-antioxidant, radical-scavenging activity, MMA-BPO, induction period, cooxidation

## Abstract

The radical-scavenging activity of dietary phytophenols has been investigated by many researches due to their antioxidant, anti-inflammatory and anticancer property but the radical-scavenging effect of 2-phytophenol and the phytophenol:co-antioxidants, vitamin C and thiol combination under nearly anaerobic conditions still remains unknown. The radical-scavenging activity for seventeen phytophenols and for six synthetic phenols (positive controls) was investigated using the induction period method in the polymerization of methyl methacrylates (MMA) initiated by thermal decomposition of benzoyl peroxide (BPO) by monitoring differential scanning calorimetery (DSC). The k_inh_ for the phytophenols was likely with the range 0.5 × 10^3^ M^−1^s^−1^−2.2 × 10^3^ M^−1^s^−1^, whereas that for synthetic phenols, hydroquinone and galvinoxyl, was with the range 7 × 10^3^ M^−1^s^−1^−8 × 10^3^ M^−1^s^−1^. Also, the additive scavenging effect of the (−)-epigallocatechin (EGC):(−)-epicatechin (EC) and the (+)-catechin:epicatechin (EC) combination was observed at 1:1 molar ratio, whereas that of the EC:quercetin combination showed the cancel (prooxidative) effect. Furthermore, the EGC:ASDB (L-ascorbyl 2,6-dibutylate) or 2-ME (2-mercaptoethanol) combination showed the prooxidative effect. Such enhancement of prooxidation in the combination may increase their toxic effects due to their cooxidation. Also, the synergic, additive or cancel effects of the flavonoid:vitamins E combination on the induction period in the BPO (a PhCOO* radical) and 2,2′-azobisisobutyronitrile (AIBN, an R* radical) systems are discussed.

## 1. Introduction

Dietary phytophenols are approximately divisible into single-ring phenolic acids such as ferulic acid, *p*-coumalic acid and caffeic acid, double-ring biphenols such as curcumin and tricyclic phenols (flavonoids) such as (+)-catechin, (−)-epicathechin (EC), (−)-epigallocatechin (EGC) and (−)-epigallocathechingallate (EGCG). They are well known to have antioxidant anti-inflammatory and anticancer properties [1]. Their probable mechanism is free radical scavenging and selective interference with various factors of abnormal proliferation of mammalian cells [1]. The phytophenols in every cup of tea or cafe may act as antioxidants to help neutralize the harmful effects of free radicals in biosystems.

Benzoyl peroxide (BPO) as an initiator is used widely for denture based resins. In combination with BPO and tertiary amines, self-curing denture-repair resins are widely used in dentistry. Currently, initiators such as camphoroquinone and benzyl as a photopolymerization system are widely used in dental materials [2]. BPO and related peroxides as an active molecule were previously reported to induce the lipid peroxidation of cellular membranes and consequently cell damage [3]. Due to the fact that dentures and restorative materials remain in the oral cavity for a very long time, BPO and related initiators may be involved in allergy and inflammatory activity [4,5,6]. Harmful free radicals derived from dental materials may be scavenged by dietary phytophenols found in food [7]. We investigated previously the radical scavenging activity of various dietary phytophenols such as polyphenols [8,9,10], single-ring phenolic acids [11], biphenols and related-phenols [12] using induction period method in polymerization of MMA initiated by BPO and/or AIBN by monitoring DSC. However, the comparative antioxidant activity for mono-ring phenols, biphenols and tricyclic phenols and for their combination was not sufficiently investigated. In the present study, we investigated the radical-scavenging activity for 13 phytophenols and butylated hydroxytoluene (BHT), a typical synthetic antioxidant, and 2-flavonoid combination and the flavonoid:co-antioxidant, ascorbate or thiol combination using the induction period method in polymerization of MMA initiated by thermal decomposition of BPO.

## 2. Results

### 2.1. Characterization of the Radical-Scavenging Activity

**Figure 1:**
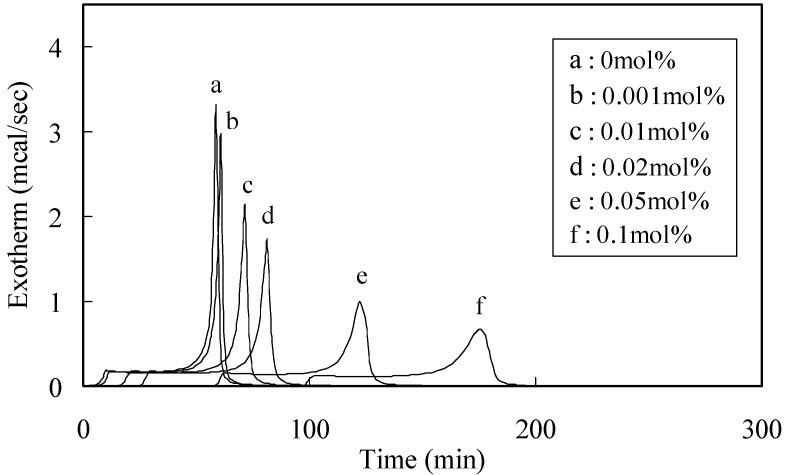
Exothermic curves for the polymerization of MMA with BPO in the presence of capsaicin.

**Figure 2:**
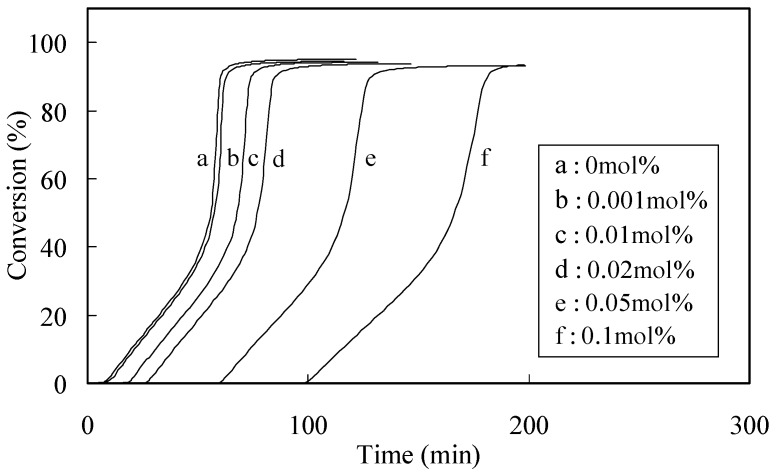
Time-conversion curves for the polymerization of MMA with BPO in the presence of capsaicin.

Polymerization curves were derived from the DSC thermogram using the integrated heat evoked by the polymerization of MMA. Typical time-exotherm and time-conversion curves for capsaicin are shown in Figure 1 and Figure 2, respectively.

**Figure 3:**
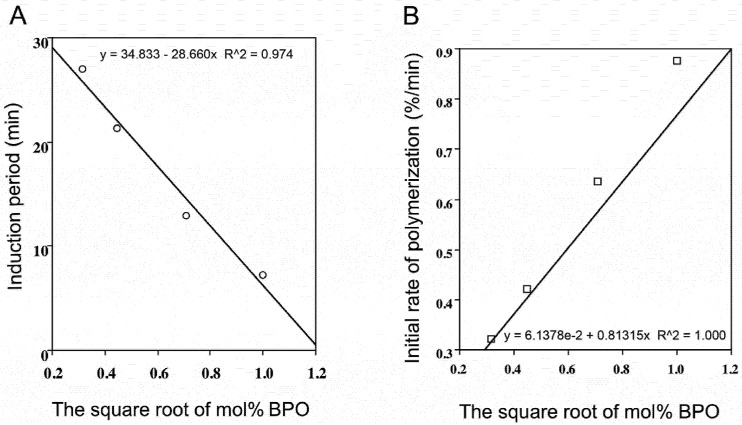
Relationships between induction period (**A**) or initial rate of polymerization (**B**) and the square root of mol% of BPO.

**Figure 4:**
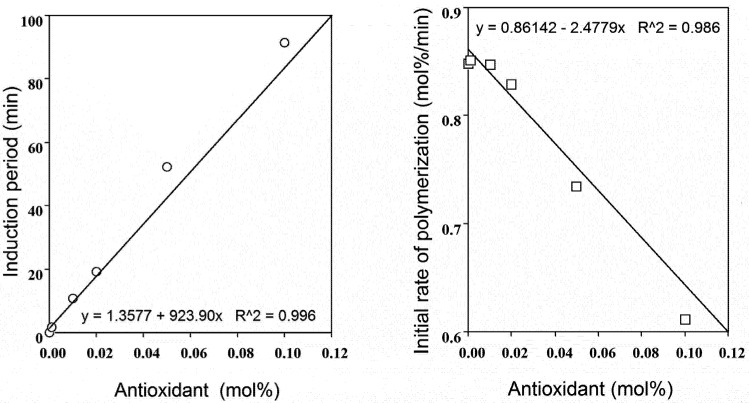
Plots of the induction period (left side panel) or the initial rate of polymerization (right side panel) against the concentrations of capsaicin.

As shown in Experimental section, using Equations (3), (6) and (7), the stoichiometric factor (*n*) (*i.e*., the number of radicals trapped by each inhibitor molecule), k_inh_ and kinetic chain length (KCL) for indicated phytophenols were determined. Results are shown in Table 1. We also examined the induction period (IP) and inhibition rate of propagation (Rp_inh_) when BPO varied with 0.1–1.0 mol% without an inhibitor. Note that a certain amount of oxygen in air was contained in the DSC sample pan and the estimated oxygen was approximately 8.128 × 10^−8^ mol/L. The relation between the induction period (A) or the initial rate of polymerization (propagation rate, Rp) (B) is shown in Figure 3 and Figure 4 respectively.

As shown in Figure 3, induction period (IP) and initial rate of polymerization (Rp) were linearly dependent o*n* thesquare rootofth*e* initiatorconcentration. Also, the Rp was proportional to the concentration of the monomer (data not shown). Thus, the kinetic studies cab be applicable using the induction period. The kinetic chain length (KCL) was calculated by equation 7. KCL of control and phenolic inhibitors at 1 mol% BPO is shown in Table 1. For all samples, the conversion rate, as calculated from the DSC thermograms, was 93.2–96.7%.

**Table 1:** Radical-scavenging activity for dietary phutophenols and synthetic phenols using induction period.

Phenolic antioxidants	Induction period method ^a)^			DPPH method ^b)^
	k_inh_ × 10^−3^			
	*n*	(M^−1^s^−1^)	KCL ^c)^	EC_50_ (mM)
A) Phytophenols				
EGC	2.6	0.85	478	0.01
EGCG	5.0	0.50	420	0.003
Eugenol	1.4	1.01	587	0.082
Caffeic acid	1.8	1.07	569	0.027
Capsaicin	0.6	1.49	570	_
Catechin	3.2	0.66	501	_
Chlorogenic acid	1.7	0.93	587	_
*p*-Coumaric acid	1.1	1.71	541	_
Curcumin	2.5	0.68	550	0.043
*trans*-Cinnamic acid	nar	nar	nar	_
Hesperetin	1.0	2.21	548	_
Isoeugenol	1.8	0.87	610	0.056
Ferulic acid	1.6	1.20	560	0.145
*n*-Propyl gallate	1.2	1.31	585	0.007
Quercetin	1.8	0.98	600	0.017
Resveratrol	2.3	0.81	567	0.11
Tetrahydrocurcumin	3.3	0.79	584	0.035
B) Synthetic phenols				
BHT	1.9	0.79	583	0.1
Bisphenol A	2.5	0.81	563	_
*p*-Cresol	1.7	1.04	487	_
DPPH	0.8	3.11	606	_
Galvinoxyl	0.3	8.24	578	_
Hydroquinone (HQ)	1.0	7.02	454	

^a)^ Described in the text; ^b)^ Anti-DPPH radical activity carried out as follows: For each phenols, various concentrations were tested in ethanol. The decrease in absorbance was determined at 517 nm for 10 min at room temperature. Antiradical activity was defined as the amount of inhibitor necessary to decrease the DPPH radical concentration by 50% (EC_50_(mol/L)). DPPH, 0.1 mM; nar: no appreciable rate.

As an example, the induction period and initial rate of polymerization for capsaicin were calculated on the base of data of Figure 2. The relationship between the induction period or the initial rate of polymerization and the concentration of capsaicin is shown in Figure 4, respectively. The induction period was linearly increased as the concentration was increased. Whereas, the initial rate of polymerization was linearly decreased as the concentration was increased.

### 2.2. Radical-Scavenging Activity

The *n* and k_inh_ value for dietary phytophenols and synthetic phenols were determined and results are summalized in Table 1. The *n* value was calculated by Equation (3). The *n* value for phytophenols declined in the order EGCG (5.0) > tetrahydrocurcumin (3.3) > catechin (3.2) > EGC (2.6) > curcumin (2.5) > resveratrol (2.3) > quercetin (1.8) > caffeic acid (1.8 ) > isoeugenol (1.8) > chlorogenic acid (1.7) > ferulic acid (1.6) > eugenol (1.4) > *n*-propyl gallate (1.2) > *p*-coumaric acid (1.1) > hesperetin (1.0) > capsaicin (0.6) > *trans*-cinnamic acid (<0.0). By contrast, that for synthetic phenols declined in the order bisphenol A (2.5) > BHT (1.9) > *p*-cresol (1.7) > HQ (1.0) > DPPH (0.8) > galvinoxyl (0.3). The formation of dimer and intermediates during the radical-scavenging for phytophenols could affect their inhibition rate constant (k_inh_). The k_inh_ (M^−1^s^−1^) for the pytophenols was as follows: the k_inh_ (M^−1^s^−1^) for phytophenols declined in the order hesperetin (2.2) > *p*-coumalic acid (1.7) > capsaicin (1.5) > *n*-propyl gallate (1.3) > ferulic acid (1.2) > caffeic acid (1.1) > eugenol (1.0) > chlorogenic acid (0.9) > isoeugenol (0.9) > quercetin (1.0) >EGC (0.9) > resveratrol (0.8) > tetrahydrocurcumin (0.8) > curcumin (0.7), catechin (0.7) > EGCG (0.5) > *trans*-cinnamic acid (<0.0). *trans*-Cinnamic acid did not inhibit the polymerization because there is no phenolic OH group in the ring. Hesperetin and *p*-coumalic acid constituted the group of the largest k_inh_ value, whereas curcumin, catechin and EGCG was a group of the smallest value. The k_inh_ for hesperetin was approximately four-fold greater than that for EGCG. EGCG, EGC and catechin, flavan derivatives were a group for the smallest KCL value of 421-501 (Table 1). Comparing their KCL to that of control, the KCL for EGCG was reduced to about 30%, whereas that for EGC and catechin was reduced to about 20%. This finding demonstrated that these compounds are the efficient chain-breaking antioxidant. Although hesperatin, *n*-propyl gallate, quercetin and resveratrol are a polyphenol group, their KCL value was 548–610, indicating that these compounds were a group of the weak chain breaking compounds and also that their activity was similar to that for monophenols, ferulic acid, eugenol, isoeugenol, chlorogenic acid and caffeic acid. On the other hand, the k_inh_ for synthetic phenols declined in the order galvinoxyl (8.2) > HQ (7.0) > DPPH (3.1) >> *p*-cresol (1.0) > bisphenol A (0.8) > BHT (0.8). Although galvinoxyl and DPPH are stable radicals, they were also a potent inhibitor with the large k_inh_ value. As apolymerization inhibitor, HQ preferably prevented polymerization of MMA, resulting from the large k_inh_ and small KCL value.

Anti-DPPH activity was shown in Table 1. The activity declined as follows: EGCG > *n-*propyl gallate > EGC > quercetin > capsaicin > tetrahydrocurcumin > curcumin> isoeugenol > eugenol > BHT> resveratrol > ferulic acid. Both EGCG and *n*-propyl gallate, containing the gallate group showed the large DPPH radical-scavenging activity.

### 2.3. Polyphenol Combinations

The result of radical-scavenging effect of the combination of EC:catechin, EC:EGC and EC:quercetin at 1:1 molar ratio is shown in Table 2.

**Table 2:** Radical-scavenging activity for 2-polyphenols combination.

	Polyphenols ^a)^	Induction period (IP)			Rp ^b)^	Conversion
		(min)	(min)		(%/min)	(%)
		Observed (A)	Calculated (B)	B/A		
	Control	6.958	_	_	0.973	95.0
	Epicatechin (EC)	35.005	_	_	0.875	93.0
	Epigallocatechin (EGC)	30.188	_	_	0.873	93.9
	Catechin	34.314	_	_	0.888	93.2
	Quercetin	25.027	_	_	0.893	94.2
	EC + Catechin	69.623	69.319	1.000	0.797	92.4
	EC + EGC	60.301	65.193	0.924	0.866	92.3
	EC + Quercetin	49.968	65.133	0.767 *	0.901	93.0

^a)^ Each 1 mM; ^b)^ Propagation rate (initial rate of polymerization). The IP is the mean of three independent experiments. The standard errors are <8%. * A *vs.* B, p < 0.01.

The B/A value at the molar ratio of 1:1 for the EC:quercetin combination was approximately 0.8, showing that when the experimental IP for the mixture was compared with the total sum of the IP of EC + quercetin, the IP decreased by approximately 20%. The radical-scavenging activity for the EC; quercetin combination balanced out. Similarly, the B/A ratio of the EC:EGC combination was approximately 0.9, showing approximately the decrease in 10%. The radical-scavenging activity for the EC:EGC combination balanced out but the activity was relatively smaller than that for the EC:quercetin combination. In contrast, that for the EC:catechin combination was 1, showing that the radical-scavenging effect for this mixture was additive.

### 2.4. Polyphenol: Co-Antioxidant Combination

Next, the radical-scavenging activity for EGC or epicatechin in combination with ASDB or 2-ME was investigated. ASDB and 2-ME were used as a biological model for vitamin C and glutathione (GSH), respectively, because water-soluble vitamin C and GSH were unable to dissolve in MMA. The result is shown in Table 3. The EGC:ASDB and the EGC:2-ME combination balanced out, *i.e*., a cancelling effect was noted.

**Table 3:** The radical-scavenging activity for EC, EGC and the EGC:EC, ASDB or 2-mercaptoethanol (2-ME) combination.

Polyphenols ^a)^	Induction period			Rp ^b)^	Conversion
	(min)	(min)		(%/min)	(%)
	Observed (A)	Calculated (B)	B/A		
Control	7.407	_	_	0.927	95.1
Epicatechin (EC)	34.028	_	_	0.866	93.4
Epigallocatechin (EGC)	26.908	_	_	0.885	93.6
ASDB	7.411	_	_	0.927	94.9
2-ME	9.218	_	_	9.218	95.0
EGC + EC	59.02	60.936	0.970	0.840	92.6
EGC + ASDB	29.612	34.319	0.860^*^	0.893	93.3
EGC +2-ME	29.373 *	36.126	0.810^*^	0.891	93.2

^a)^ Each 1 mM, ^b)^ propagation rate (initial rate of polymerization), ASDB (*L*-ascorbyl 2,6-dibutylate). The values are the mean of three independent experiments. The standard errors are <10%. *A *vs.* B, p < 0.01.

## 3. Discussion

Previously quantitative *in vitro* studies of the radical-scavenging activity of Burton and Ingold reported previously the kinetics of radical-scavenging activity of phenolic compounds in the chlorobenzene-styrene system initiated by azobisisobutyronitrile (AIBN) at 30 °C using induction period method [13]. In this method, autooxidations were carried out under 760 torr of O_2_ in an automatic recording gas pressure transducer apparatus. Styrene has no abstractable hydrogens and forms a polymeric peroxyl radical. The end of inhibition period can be calculated by inhibited oxygen —time curves on the base of the interception of a base and initial lines. By contrast, we investigated the kinetics of radical-scavenging activity of phenolic compounds in polymerization of MMA initiated by thermal decomposition of AIBN and/or BPO using differential scanning calorimetry (DSC) [8,9,10]. The model using induction period method was able to explain the mechanism of radical-scavenging activity and to predict the chain-breaking activity of phenolic compounds, because measurement by DSC is highly sensitive. Also, the DSC system in the present study was performed by the induction period method under aerobic conditions. The oxygen tension under a 15 torr oxygen atmosphere is similar to that in many tissues [14,15], suggesting that oxygen is scarce in living cells and that the radical-scavenging activity of phytophenols *in vivo* may differ considerably from that observed under aerobic conditions.

The *n* value for EGCG was the greatest among the compounds tested, followed by EGC and catechin (Table 1). As shown in Table 2, the radical-scavenging activity for EGC, catechin, EC and quercetin was investigated and it was found that the *n* value for EGC, EC, catechin and quercetin was 3.15, 3.83, 3.69 and 2.46, respectively. EC and catechin showed a similar value to that shown in Table 1. The antioxidant effect of (+)-epimer, catechin and (−)-epimer, EC was similar. The inhibitory effect of catechins on peroxidation of soybean phosphatidylcholine liposomes was investigated by 2,2′-azobis(2-aminopropane)hydrochloride (AAPH)-induced oxidation and it was found that the *n* value for EC, EGC and EGCG was 3.2, 1.1 and 1.7, respectively [16]. The *n* value for EGC and EGCG, particularly the latter, was less than that in the present study. This may be depend on the experimental conditions. In general, the *n* value of monophenols is 2. The *n* value for caffeic acid, ferulic acid and BHT is near 2. That for *p*-coumaric acid, hesperetin, eugenol and *n*-propyl gallate it is near 1, suggesting the formation of dimer derived from the monomer-monomer coupling reaction due to radical oxidation [8,11]. Also, HQ with the *n* value of 1 may undergo dimerization. Tricyclic phenols (flavonoids) such as catechin, EGC and EGCG showed the large *n* value due to their large number of phenolic OH groups.

Javanovic *et al.* previously investigated oxygen radical-scavenging activity for flavonoids and it was found that their inhibition rate of super oxygen radical (k_inh_) at 20 °C was with the range 3 × 10^2^ M^−1^s^−1^–5 ×10^4^ M^−1^s^−1^ [17]. Zhou *et al*. previously reported that the green tea polyphenols could reduce α-tocopheroxyl radical to regenerate α-tocopherol with rate constants of 0.45, 1.11, 1.31, 1.91, and 0.43 ×10^2^ M^−1^ s^−1^ for EC, EGC, ECG, EGCG, and gallic acid (GA), respectively, in sodium dodecyl sulfate micelles [18]. In the present study, the k_inh_ (M^−1^ s^−1^) for green tea polyphenols, EGCG, ECG and catechin was 0.5 × 10^3^, 0.85 × 10^3^ and 0.66 × 10^3^, respectively. That for *p*-coumalic acid and hesperatin was approximately two-fold greater than that for EGCG or EGC. Although under different experiment conditions, the k_inh_ value for catechin polyphenols was different, the difference in the relative k_inh_ between polyphenols fell with a range of several-fold. EGCG, EGC, catechin with the small k_inh_ showed their small KCL value. From the present finding of the k_inh_, EGCG scavenged much free radicals but released them at relatively short time. EGCG was found to be potent chain-breaking antioxidant, resulting from the small KCL.

There are many radical-scavenging methods for polyphenols [19]. Their scavenging effect on O_2_^−^ declined in the order EGCG > ECG > EC ≈ EGC [19]. Tea catechins are effective 1,1′-diphenyl-2-picrylhydrazyl (DPPH) antagonists. Anti-DPPH activity was EC < (+)-catechin < EGC < EGCG [20]. The *n* value for tea catechins and related compounds was investigated by the DPPH method and indicated that for catechin, EC, EGC, ECG and EGCG they was estimated to be 2, 2, 5, 7 and 10, respectively [21]. The *n* for EGC and EGCG was calculated from the data in Table 1, and it was found that their value was similar to that for above described report [21]. Anti-DPPH activity for the gallated catechins, EGCG and *n*-propyl gallate was greater than that for nongallated catechins, EGC. The antioxidant activity for tea polyphenols is also radical-dependent and medium dependent. In general, EGCG has been recognized as a potent radical scavenger.

The synergic scavenging effect of two-catechins combinations on free radicals such as O_2_^−^ was investigated, indicating that the effect of the EGCG:ECG combination was the strongest, followed by the ECG:EC and the EC:EGC combination. [19]. In the present study, however, no synergic scavenging effect of the EGC:EC and the EC: (+)-catechin combination, was found. By contrast, a prooxidative (cancelling) scavenging effect of the EGC:ASDB or 2-ME combination was observed. Also, the prooxidative effect of the EC:quercetin combination was observed. The synergistic antioxidant mechanism of α-tocopherol with green tee polyphenols, EC, EGC, ECG, EGCG and GA was previously reported and it was found that these polyphenols could reduce the α-tocopheroxy radical to regenerate α-tocopherol [18,22,23]. Also, the flavonoid:vitamin C combination caused a synergic antioxidant effect in an *in vitro* lipoprotein oxidation model [24]. We previously investigated the free radical interaction between α-tocopherol or ASDB and methyl gallate (MG), EC, EGC, ECG or EGCG. This showed a synergic radical-scavenging effect of the α-tocopherol:MG, EC, EGC or ECG combination on the induction period in the polymerization of MMA initiated by thermal decomposition of AIBN [9]. Whereas, there was no synergic scavenging effect of the ASDB:MG, EC, EGC or ECG combination and conversely, the prooxidative scavenging effect was observed for their combination [9].

On the other hand, using the induction period method initiated by BPO, a synergic radical-scavenging effect of the δ-tocopherol:EC or EGCG combination alone was observed, whereas a prooxidative radical-scavenging effect of the α-, β- or γ-tocopherol:EC or EGCG combination was observed. Also, the ASDB:EC or EGCG combination showed a prooxidative radical-scavenging effect [10]. The radical-scavenging effect of the mixture of flavonoid and vitamin E showed clearly the great contradictory difference between the AIBN and BPO systems. These findings may be involved in the induced decomposition of BPO. Frank *et al.* reported that dietary flavonoids, quercetin, catechin and epicatechin increase α-tocopherol in rat and protect the vitamin from oxidation *in vitro* [23]. Also, Wiegand *et al*. reported that dietary flavonoids, quercetin and catechin do not affect vitamin E status in growing rats [24]. These contradictory findings suggested that the vitamin E status in the presence of flavonoids may be dependent on radical species.

In the previous our studies, a synergic radical scavenging effect of the vitamin E:flavonoid combination was found under R* radical conditions derived from the AIBN system [9]. This suggested that the antioxidant activity *in vivo* due to bioactive compound such as vitamins and GSH ocurrs under nearly anaerobic conditions because biological systems tend to have low oxygen tension [14,15]. Interestingly, the radical-scavenging effect of the flavonoid:vitamin C combination oxidized by both radicals R* and PhCOO* was the prooxidative one [9,10]. That of the EGC:2-ME combination was also prooxidative (Table 3). The radical-scavenging effect of the EC:2-ME combination oxidized by both radicals R* and PhCOO* was the prooxidative (data not shown). Green tea polyphenols are well-known to possess antioxidant and anticancer activity. Cancer cells are anaerobic in the metabolism and have very poor mechanisms for absorbing adequate amounts of antioxidants [25,26]. Dietary phytophenols not only interact directly with free radicals, but also can react with other redox-based antioxidant substances. These redox cycles of vitamin E, vitamin C or GSH form a well-known antioxidant network. Such antioxidant network could prevent chronic inflammation and cancer due to free radical reactions in biological systems. Further studies may be necessary to clarify the radical-scavenging effect of phytophenols in the presence of vitamin C and vitamin E and GSH. On the other hand, the inhibition of polymerization for phenolic inhibitors is very important in the chemical industry. In the present study, HQ in addition to galvinoxyl showed one-order grater k_inh_ value than that for EGC and EGCG and its KCL was the smallest (454). We have seen HQ as a potent inhibitor, in the fresh light.

*In vivo* experiments are too complex to amenable to simple interpretation and, hence, we employed physical-chemical studies using the induction period method in the radical polymerization of MMA under nearly anaerobic conditions. We expect that induction period, *n*, kinh, KCL values for dietary phytophenols determined in the present study will be relevant for the development of compounds that mimic their biological activity.

## 4. Experimental

### 4.1. Materials

The following chemicals were obtained from the indicated companies: (+)-catechin, quercetin, hesperetin, resveratrol (Sigma Chemical Co., St. Louis, MO, USA). (−)-Epigalloctechin gallate (EGCG), (−)-epicatechin, (−)-epigallocatechin (Kurita Analysis Service Co. Ltd., Tsukuba, Japan). Bisphenol A, 2,6-di-*tert*-butyl-4-methylphenol (BHT), *trans*-cinnamic acid, caffeic acid, *p*-coumalic acid, curcumin, *p*-cresol, chlorogenic acid, 1,1-diphenyl-2-picrylhydrazyl (DPPH), eugenol, ferulic acid, gaivinoxyl, hydroquinone (HQ), isoeugenol, BPO and MMA (Tokyo Kasei Co. Japan). MMA was purified by distillation. BPO was crystallized from chloroform/methanol (1:1) solution. Tetrahydrocurcumin was kindly donated by Dr. I. Yokoe of Josai University.

### 4.2. Methods

The experimental resin consisted of MMA and BPO with or without phenolic antioxidants. BPO were added at 0.1, 0.2, 0.5 and 1.0 mol% and additives were used at 0.001, 0.01, 0.02, 0.05 and 0.1 mol% when BPO was 1.0 mol%. The combination study was carried out for 0.01 mol% for each phenol with 1.0 mol%. Approximately 10 µL of the experimental resin (MMA: 9.12–9.39 mg) was loaded into an aluminum sample container and sealed by applying pressure. The container was placed into a different scanning calorimeter (model DSC 3100: Mac Science Co., Tokyo, Japan) maintained at 70 °C, and the thermal changes induced by polymerization were recorded for appropriate time periods. Exothermic curves for the polymerization of MMA with BPO in the presence of capsaicin, a typical compound is shown in Figure 1. The heat produced due to polymerization of MMA was 13.0 kcal/mol in these experiments.

Polymerization curves break when an inhibitor is consumed (Figure 2). These breaks are sharp and provided a reliable measure of the induction period of the antioxidant inhibitor. The presence of oxygen retards polymerization because oxygen reacts with MMA radicals (MMA*) activated by the initiator, and the produces a non-radical product. Thus, polymerization of the control was slightly inhibited, even though the reaction was carried out in a sealed DSC pan, because the pan contained a small amount of oxygen since it had been sealed in air. At an early stage in each run, tangents were drawn to the polymerization curves. The induction period for each test compound was determined from the length of time between the zero point on the abscissa and the point of intersection of the tangent with the polymerization curve. The induction period (IP) was calculated at the difference between the induction period of the specimen and that for the control. The initial rates of polymerization in the absence (Rp_con_) and presence (Rp_inh_) of the phytophenols were calculated from the slope of the first straight line on each plot of the conversion rate during MMA polymerization [8]. The Rp_inh_ value represents the rate of inhibition of initial polymerization by the antioxidant.

### 4.3. Measurement of Rate of Initiation

Initiation of BPO, an initiator:


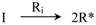
(1)

The induction period method was also used to determine the rate of initiation of polymerization (R_i_) due to thermal decomposition of BPO using in Equation (1):

R_i_ =*n*[IH]_0_ / IP (2)

in which [IH]_0_ is concentration of the initiator at time zero and the IP is the induction period. DTBMP was used to determine R_i_, since the stoichiometric factor, *n*, is known to be 2.00. In the case of MMA = 9.4 M and BPO = 0.1 M at 70 °C gave an R_i_ value of 2.28 × 10^−6^ M s^−1^.

### 4.4. Measurement of Stoichiometric Factor (n)

The relative n value in Equation (3) can be calculated from the induction periods in the presence of inhibitors.

*n* = R_i_[IP] / [IH] (3)

where [IP] is the induction period in the presence of an inhibitor. The number of moles of peroxy radicals trapped by the relavant phenol is calculated with respect to 1 mole of inhibitor moiety unit.

### 4.5. Measurement of the Inhibition Rate

When R_i_ is constant, *i.e*., when new chain are started rata constant rate, a steady-state treatment can be applied and the initial rate of polymerization of MMA is given by Equation (4):

Rp = {k_p_[MMA]Ri^1/2^} / (2k_t_)^1/2^ (4)

where MMA represents methyl methacrylates and k_p_ and k_t_ are the rate constants for chain propagation and termination, respectively. The k_p_/(2k_t_)^1/2^ rate of polymerization of MMA (9.4 M) by BPO (0.1M) at 70 °C was 9.86 × 10^−2^M^−1/2^ s^−1/2^ [8]. The k_t_ was estimated to be approximately 3.8 × 10^7^ and therefore, the k_p_ was approximately 930.

Rp_inh_ = (k_p_[MMA]R_i_) / (*n* k_inh_[IH]) (5)

in which is the initial rate ofpolymerization with an inhibitor. [MMA], *n*, [IH] and kp are defined above and kinh is the rate constant for scavenging (inhibiting) of MMA radicals by a phytophenol antioxidant.

Equations (3) and (5) give Equation (6):

k_inh_/k_p_ = [MMA]/(Rp_inh_[IP]) (6)

The kinetic chain length is given by Equation (7):

KCL = Rp_inh_ /R_i_ (7)

## 5. Conclusions

The radical-scavenging activity in the polymerization of MMA initiated by thermal decomposition of BPO under nearly anaerobic conditions was investigated by the induction period method for thirteen dietary phytophenols and one artificial phenol (BHT). The stoichiometric factor (*n*), k_inh_, KCL for these compounds was determined. Their anti-DPPH activity was also determined. The radical-scavenging effect of the EC:catechin, EGC or quercetin combination and the EGC:ASDB or 2-ME combination at molar ratio 1:1 were investigated. The EGC:quercetin, ASDB or 2-ME combinations showed prooxidative effects. The synergistic, additive and cancelling (prooxidative) radical-scavenging effects of the combination were discussed.
